# Delayed Diagnosis of a Diffuse Invasive Gastrointestinal Aspergillosis in an Immunocompetent Patient

**DOI:** 10.1155/2020/3601423

**Published:** 2020-05-23

**Authors:** S. Guglielmetti, C. M. Jaccard, K. Mühlethaler, A. Bigler, D. Springe, L. Ebnöther, M. Delgado

**Affiliations:** ^1^Emergency Department, Bürgerspital Solothurn, Switzerland; ^2^Institute of Pathology, University of Bern, Switzerland; ^3^Institute for Infectious Diseases, University of Bern, Switzerland; ^4^Intensive Care Unit, Anesthesiology Department, Bürgerspital Solothurn, Switzerland

## Abstract

Invasive aspergillosis represents a clinical picture frequently associated with host's immunosuppression which usually involves a high morbidity and mortality. In general, the most frequent fungal entry is the lungs with secondary hematogenous dissemination, but there are other hypotheses like a gastrointestinal portal of entry. There are some rare publications of cases with invasive aspergillosis in immunocompetent patients. We present the case of an immunocompetent patient without any risk factors except for age, ICU stay, and surgical intervention, who developed a septic shock by invasive gastrointestinal aspergillosis as primary infection. Due to the unusualness of the case, despite all the measures taken, the results were obtained postmortem. We want to emphasize the need not to underestimate the possibility for an invasive aspergillosis in an immunocompetent patient. Not only pulmonary but also gastrointestinal aspergillosis should be taken into account in the differential diagnosis to avoid a delay of treatment.

## 1. Introduction

Aspergillus represents a group of fungi with a ubiquitous distribution, whose spores (conidia) are easily transported through the air, as most important transmission route. Within the group of aspergillus, A. fumigatus represents more than 80% of severe human aspergillus infections [1]. The most frequent human infection is invasive pulmonary aspergillosis, followed by infections of nasal sinuses, the central nervous system, and, rarely, gastrointestinal infections. Ingested spores cannot penetrate an intact mucosal barrier, but pathology of the mucosal barrier is needed to cause an invasive gastrointestinal aspergillosis [2, 3].

Patients affected by aspergillus are generally immunocompromised, with chronic use of corticosteroids or immunosuppressive treatments, prolonged neutropenia, hepatic cirrhosis, chronic granulomatous diseases, or a human immunodeficiency virus infection. Diabetics with hyperglycemia also have an impaired immune response, which permits bacterial or fungal colonization and sometimes invasion through the skin or mucosa [1, 4]. It is believed that these patients show an impaired immune response in mononuclear phagocytic function, leading to a dysfunction of alveolar macrophages [5]. An invasive aspergillosis in these patients presents as a severe illness with an unfavorable prognosis. We are reporting a rare case of an invasive gastrointestinal aspergillosis in an immunocompetent patient without any risk factors. The outcome in this case is equally bad and illustrates why early recognition and intervention is crucial for the prognosis.

## 2. Case Presentation

In the year 2019, an 88-year-old, autonomous female with a past medical history of hyperparathyroidism, dyslipidemia, and nonrelevant cardiopathy (treatment with aspirin, metoprolol, and atorvastatin) presents in the emergency room with vomiting and diarrhea after eating fish the day before. For months, she suffered from diffuse abdominal pain, for which she medicated herself with paracetamol and metamizole. At the first examination, the patient was in a good general condition but showed an erythematous and painful left arm. The blood test showed leukocytosis (7.3 G/l) with left deviation and C-reactive protein (CRP) of 175 mg/l. An antibiotic therapy with ceftriaxone intravenous was begun, and an outpatient treatment was started with daily consultation for medication administration. At the second consultation, the patient showed a deterioration of general state, with disorientation, slowing down, and tendency to hypotension. The left arm showed an aggravation with swelling, livid discoloration of the skin, compatible with erysipelas. The abdomen was distended, painful, without peristalsis. A reexamination of the blood indicated an acute renal failure (creatinine 279 *μ*mol/l), CRP 436.4 mg/l, and rhabdomyolysis (creatinine kinase (CK) 12982 U/l) with metabolic acidosis (pH 7.26, PaO_2_ 10.9 kPa, PaCO_2_ 3.7 kPa, HCO_3-_ 12.3 mmol/l, and BE -15 mmol/l with lactate 2.8 mmol/l). An urgent computer tomography (CT) showed unspecific diffuse intestinal dilatation without signs of perforation or free fluid. The lungs presented only with dorsal right dystelectasis but revealed no pulmonary infiltrations. Due to the rapid clinical deterioration, it was decided to take blood cultures, begin with antibiotics (piperacillin-tazobactam, clindamycin), and transfer the patient to the intensive care unit with the diagnosis of sepsis due to erysipelas of the left arm with suspected fasciitis.

On arrival at the intensive care unit, the patient was increasingly agitated and an intravenous therapy with dexmedetomidine was initiated. To restore hemodynamic stability, a combined therapy with fluids and noradrenalin was started. Three liters of oxygen through a nasal cannula was sufficient to achieve a good oxygenation of the lungs. However, there was a progressive distension of the abdomen and a clinical deterioration with no clear focus of infection, resulting in an exploratory laparotomy. Intraoperatively, a puncture of the joints and tissue biopsies of the left arm and hand were performed with negative results in the cultures the following days. Intra-abdominally, there were multiple tissue adhesions with signs of low vascular perfusion, but no signs of spontaneous intestinal perforation. Because of an accidental colon injury and observed general low tissue perfusion, it was decided to conduct a split colostomy. The following days, the patient remained under mechanical ventilation with hemodynamic instability and agitation. Vasoactive drugs could be reduced, and lactate as well as inflammatory parameters improved. A week after surgery, the stoma was covered with a large quantity of filamentous structures compatible with fungal colonization. After collecting surface cultures, a local treatment with amphotericin B was begun. The following day, the same growth was found in the stoma. Simultaneously, the clinical status rapidly declined with an increase of inflammation parameters and a new deterioration of renal function, which required an increase of vasoactive drugs. Since the patient had an advance directive limiting therapeutic measures in the case of severe illness or need of renal substitution, a joint decision with the family was taken to initiate comfort therapy. The patient died a few hours later, after a week spent in the intensive care unit.

**Figure 1:**
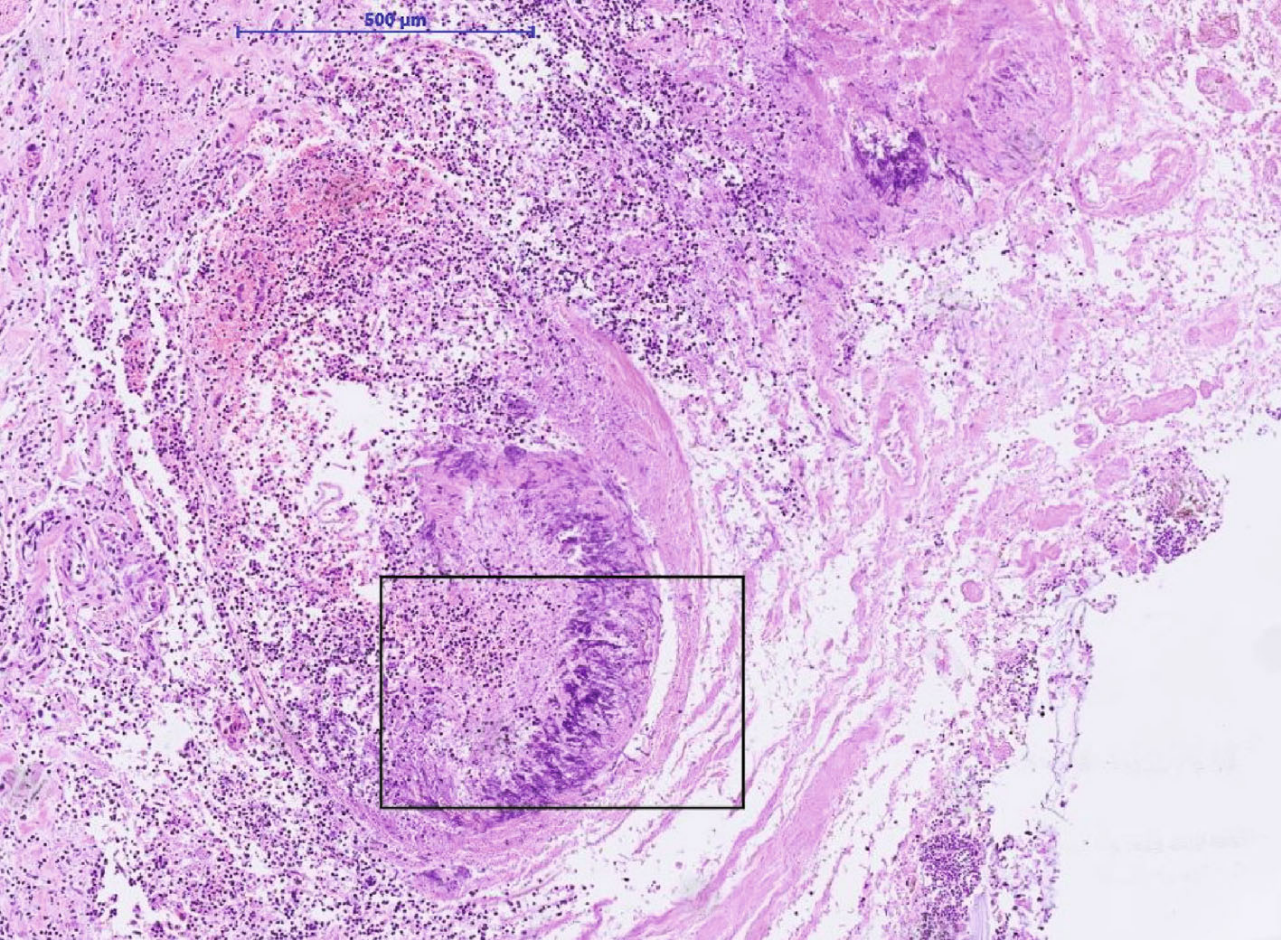
Hematoxylin-eosin staining of ulcers of the small intestine with intravascular aspergillus manifestation (4x magnification).

**Figure 2:**
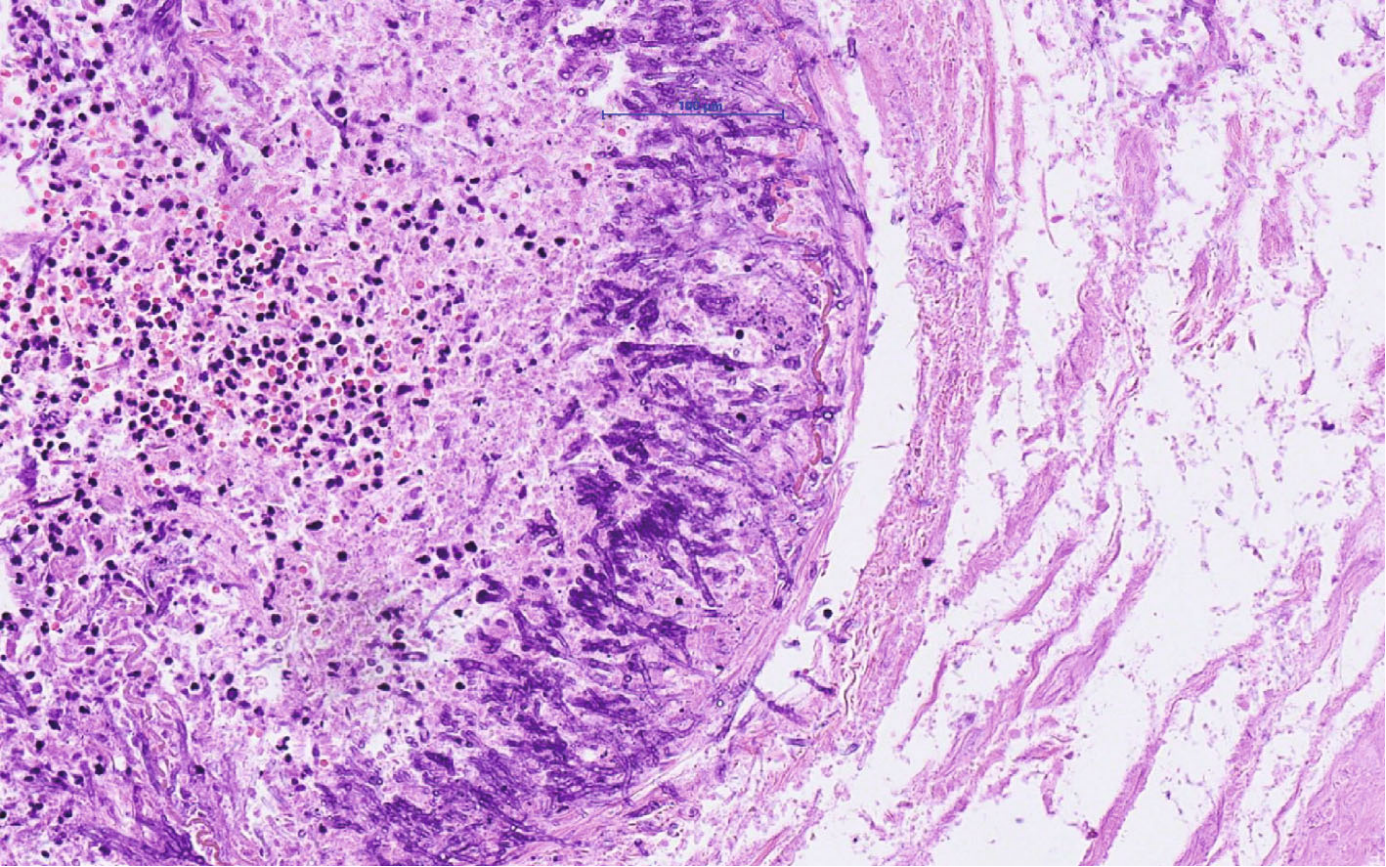
Higher magnification (20x) of marked area in the image.

**Figure 3:**
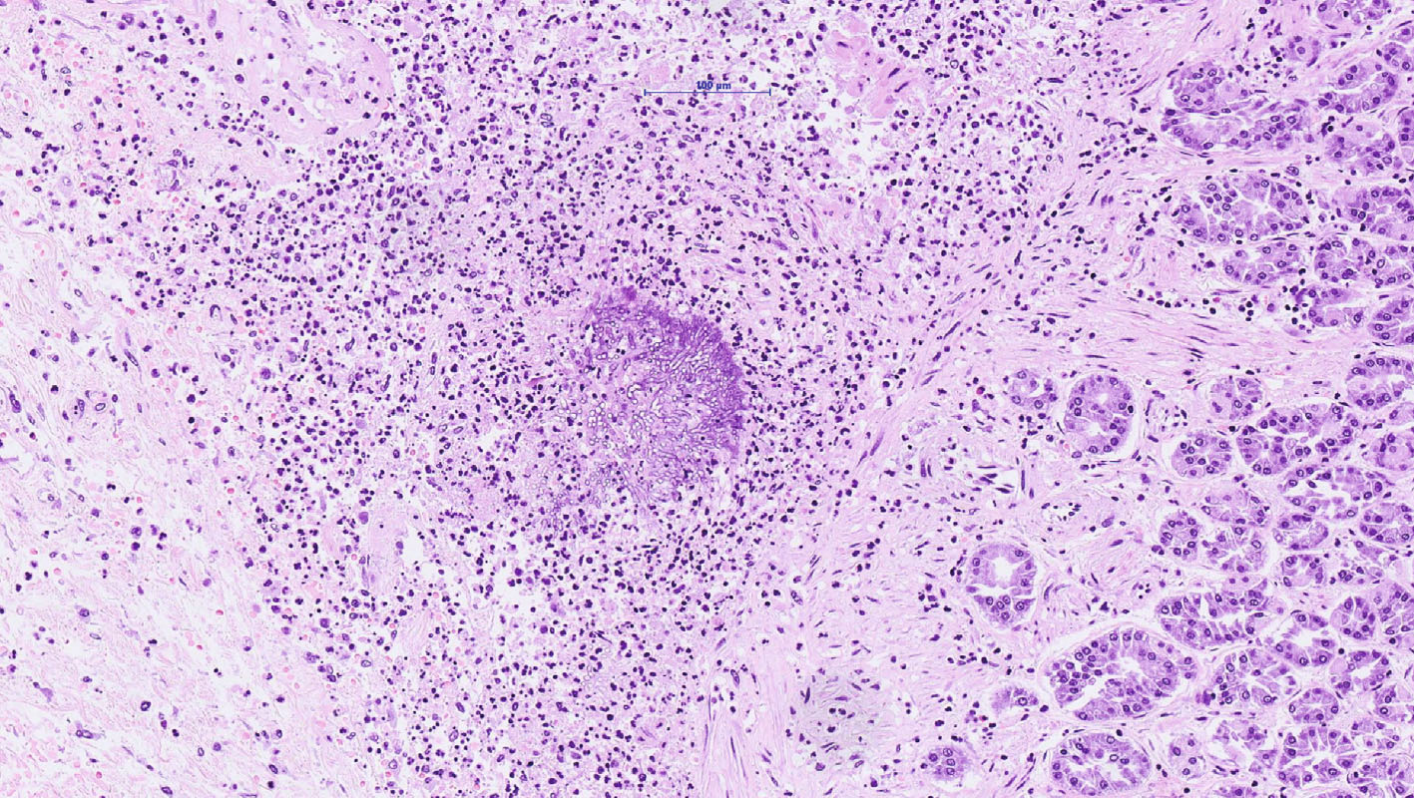
Hematoxylin-eosin staining of the stomach with submucosal aspergillus (20x magnification).

**Figure 4:**
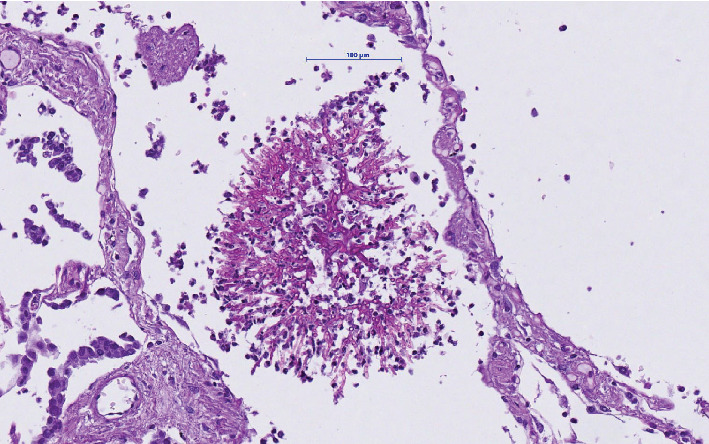
Periodic acid-Schiff reaction of aspergillus in the right lower lung lobe (20x magnification).

The microbiologic culture showed the presence of Aspergillus fumigatus in the stoma. Because of a fulminant infectious process with unknown focus, the permission to perform an autopsy was obtained from the family. Postmortem investigation showed multiple gastric and intestinal ulcerative lesions with evidence of an invasive fungal infection. Fungi could be detected within the mucosa and mucosal and submucosal vessels. By morphology and also confirmed by molecular pathology, the presence of Aspergillus fumigatus was confirmed (Figures 1–3). The presence of fungi could also be demonstrated in the lungs, in particular the alveoli of the right lower lobe; however, it was without signs of invasion (Figure 4). The biopsies of the left arm showed a phlegmonous inflammation without mycotic presence.

## 3. Discussion and Conclusions

The diagnosis of a fungal infection keeps being a challenge: the symptoms of gastrointestinal aspergillosis, such as abdominal pain, diarrhea, hemorrhage, and possibly intestinal obstruction with perforation, are nonspecific, so a high clinical suspicion is needed to consider testing for aspergillosis (for example, by testing for antigens like galactomannan [6] and beta (1, 3)-D-glucan [7]). One of the most extensive descriptions of invasive aspergillosis is the work of Eggimann et al. [2]. Their review of gastrointestinal aspergillosis reports only immunocompromised patients; most of them were disseminated but extremely rarely caused by isolated gut aspergillosis (8%). The authors propose to consider not only an air portal entry but also a gastrointestinal portal entry for invasive Aspergillosis spp. They postulate that aspergillus spores can find favorable conditions to reproduce in the ulcers on the mucous surface of the digestive tract. In our case, at postmortem diagnostics, we found aspergillus in the alveoli and trachea, but could not prove pulmonary invasion histologically despite extensive tissue sampling. We therefore interpret their occurrence as secondary (possibly due to aspiration). The partial colectomy specimen submitted after the intraoperative injury showed ischemic mucosal injury, but no fungi could be objectified even after the second review. Still, a contamination during surgical intervention and accidental perforation cannot be excluded. In contrast, the patient came to the hospital with gastrointestinal symptoms and a long history of abdominal pain. It seems plausible that the ulcerative mucosal lesions caused by a general ischemic status throughout the entire intestinal tract provided the base for mycotic invasion, in line with Eggimann's hypothesis. To the best of our knowledge, this constellation of primary gastrointestinal aspergillosis in an immunocompetent patient without risk factors has been described in only a handful of articles in the literature so far [3]. The retrospective study of Kazan et al. [8] analyzes the presence of gut aspergillosis in 21 patients with leukemia and allogenic hematopoietic stem cell transplant. They describe poorly specific symptoms like a prolonged history of abdominal pain, diarrhea, hemorrhage, and intestinal occlusion or perforation. In some cases, a diagnosis was obtained through endoscopy and biopsy with similar findings to ours. Eight patients presented with isolated gastrointestinal aspergillosis which postulates an oral portal of entry due to the ingestion of contaminated food. In this series, the reported mortality was 57% and an optimal therapy (surgery, antifungals, or combined) could not be established. In the same line, the review of Reischies and Hoenigl investigates the role of surgery as adjunctive option to antifungal therapy in different manifestations of invasive aspergillosis [9]. Despite all current therapeutic options, the mortality rate of this opportunistic infection remains high as described by Denning [10].

Our report illustrates the case of an immunocompetent patient that developed a very rare entity which led to her death. These findings led us to the conclusion that aspergillosis infection should not be underestimated in the differential diagnosis, even in immunocompetent patients.

## Abbreviations

CRP: C-reactive protein
CK: Creatinine kinase
kPa: Kilopascal
PaO_2_: Arterial partial pressure of oxygen
PaCO_2_: Arterial partial pressure of carbon dioxide
HCO_3-_: Bicarbonate
BE: Base excess
CT: Computer tomography.
